# Is leg compression beneficial for alpine skiers?

**DOI:** 10.1186/2052-1847-5-18

**Published:** 2013-09-02

**Authors:** Billy Sperlich, Dennis-Peter Born, Mikael Swarén, Yvonne Kilian, Björn Geesmann, Matthias Kohl-Bareis, Hans-Christer Holmberg

**Affiliations:** 1 Department of Sport Science, University of Wuppertal, Fuhlrottstraße 10, Wuppertal 42119, Germany; 2 Institute of Training Science and Sport Informatics, German Sport University, Cologne, Germany; 3 Department of Health Sciences, Swedish Winter Sports Research Centre, Mid Sweden University, Östersund 83125, Sweden; 4 RheinAhrCampus, University of Applied Sciences Koblenz, Suedallee 2, Remagen, 53424, Germany

**Keywords:** Balance, Blood lactate, Downhill skiing, EMG, Heart rate, NIRS, Oxygen uptake

## Abstract

**Background:**

This study examined the effects of different levels of compression (0, 20 and 40 mmHg) produced by leg garments on selected psycho-physiological measures of performance while exposed to passive vibration (60 Hz, amplitude 4-6 mm) and performing 3-min of alpine skiing tuck position.

**Methods:**

Prior to, during and following the experiment the electromygraphic (EMG) activity of different muscles, cardio-respiratory data, changes in total hemoglobin, tissue oxygenation and oscillatory movement of *m. vastus lateralis*, blood lactate and perceptual data of 12 highly trained alpine skiers were recorded. Maximal isometric knee extension and flexion strength, balance, and jumping performance were assessed before and after the experiment.

**Results:**

The knee angle (−10°) and oscillatory movement (−20-25.5%) were lower with compression (*P* < 0.05 in all cases). The EMG activities of the *tibialis anterior* (20.2-28.9%), *gastrocnemius medialis* (4.9-15.1%), *rectus femoris* (9.6-23.5%), and *vastus medialis* (13.1-13.7%) muscles were all elevated by compression (*P* < 0.05 in all cases). Total hemoglobin was maintained during the 3-min period of simulated skiing with 20 or 40 mmHg compression, but the tissue saturation index was lower (*P* < 0.05) than with no compression. No differences in respiratory parameters, heart rate or blood lactate concentration were observed with or maximal isometric knee extension and flexion strength, balance, and jumping performance following simulated skiing for 3 min in the downhill tuck position were the same as in the absence of compression.

**Conclusions:**

These findings demonstrate that with leg compression, alpine skiers could maintain a deeper tuck position with less perceived exertion and greater deoxygenation of the *vastus lateralis* muscle, with no differences in whole**-**body oxygen consumption or blood lactate concentration. These changes occurred without compromising maximal leg strength, jumping performance or balance. Accordingly, our results indicate that the use of lower leg compression in the range of 20-40 mmHg may improve alpine skiing performance by allowing a deeper tuck position and lowering perceived exertion.

## Background

As a consequence of the uneven surfaces and high speeds involved in alpine downhill skiing, the athletes are subjected to strong passive vibrations and shocks [1,2]. Displacement of a muscle by passive vibration stimulates the muscle spindles via the polysynaptic vibration reflex [3,4]. Consequently, a larger number of motoneurons [5] are activated, which elevates the electromyographic (EMG) activity of the *vastus lateralis* muscle [6] and, finally, causes early onset of muscle fatigue [7]. Moreover, pronounced oscillatory displacement of a muscle interferes with neurotransmission and the recruitment of muscle fibers during contraction in response to excitation [8]. Thus, in connection with sports associated with such displacement, such as alpine skiing, reduction of passive vibration should postpone the onset of muscle fatigue by reducing physiological, biomechanical and perceptual strain.

One way to reduce oscillatory displacement is by compressing the belly of the muscle [9]. Compression garments attenuate longitudinal and anterior-posterior oscillation of muscles during maximal jumping, thereby lowering fatigue and elevating height during consecutive jumps [9,10]. Since compression garments activate tactile mechanoreceptors, reduce pre-synaptic inhibition [11] and enhance sensory feedback, proprioception is enhanced [12,13] and balance improved [13]. In addition, compression stockings enhance maintenance of leg power after 10 km of running [14]. In connection with alpine skiing maintenance of balance [15] and the ability to generate large forces [16] are important to success and any improvements in these parameters should be beneficial [16,17]. Furthermore, compression of the thigh muscles reduces the cross-sectional area of the venous systems [18], thus improving venous hemodynamics in resting or exercising limbs ([19-22]; see Born, Sperlich and Holmberg [23] for a description of the detailed mechanism involved).

Depending on the velocity, skiing equipment and surface conditions, the frequency of ground reaction forces encounted while alpine skiing may vary considerably [2,24], ranging from 20-200 Hz during high-speed turns on hard snow [24]. Attenuation of fiber recruitment and enhancement of micro-vascular blood flow and oxygenation within the leg muscles by compression may reduce the cardio-respiratory and neuro-metabolic effort required (e.g., heart rate, oxygen uptake, muscle activity and blood lactate concentration) and thereby help to maintain jumping capacity and balance. However, at present, the influence of compressing the legs of alpine skiers while in the downhill tuck position, as well as the eventual underlying mechanism(s), remain unclear.

Accordingly, the primary goal of this investigation was to evaluate the effects of different levels of compression on the legs of highly trained alpine skiers subjected to passive vibration in the downhill tuck position. Our hypothesis was that such compression would 1) reduce oscillatory displacement of the thigh muscle; 2) lower muscle activation; 3) improve oxygenation of the *vastus lateralis* muscle; and 4) maintain maximal isometric leg force and jumping height and balance, both before and after being subjected to passive vibration.

## Methods

### Subjects and ethical statement

All participants were informed about the purpose, nature and potential risks of the study and gave their written informed consent prior to participating. All procedures were approved by the ethics committee of the German Sport University, Cologne, Germany and conducted in accordance with the Declaration of Helsinki.

### The preliminary test

In a preliminary test, the compression exerted on the calf and thigh by the suits worn during slalom and giant slalom racing by seven elite male members (24 ± 3 yrs; height: 172 ± 4 cm; body mass: 68 ± 6 kg (means ± SD)) of the German and Swedish alpine ski teams was determined 5 times employing a pneumatic sensor (SIGaT®, Ganzoni-Sigvaris, St. Gallen, Switzerland) in accordance with international recommendations [25] and as described previously [26,27]. These measurements were used to set the level of compression (moderate or high) during the subsequent laboratory testing.

### Subjects

The twelve competitive elite male alpine skiers (age: 26 ± 4 yrs; height: 178 ± 4 cm; body mass: 80 ± 5 kg (means ± SD)) who participated in this study were all familiar with the laboratory procedures involved. The criteria for inclusion were 1) membership in a national and international racing team; 2) > 10 hrs of physical training per week; and 3) participation in a minimum of 10 competitive races per year. The athletes were instructed to remain adequately hydrated and to refrain from consuming alcohol or caffeine for 24 h and food for 3 h prior to each test.

### The experimental laboratory protocol

**Figure 1:**
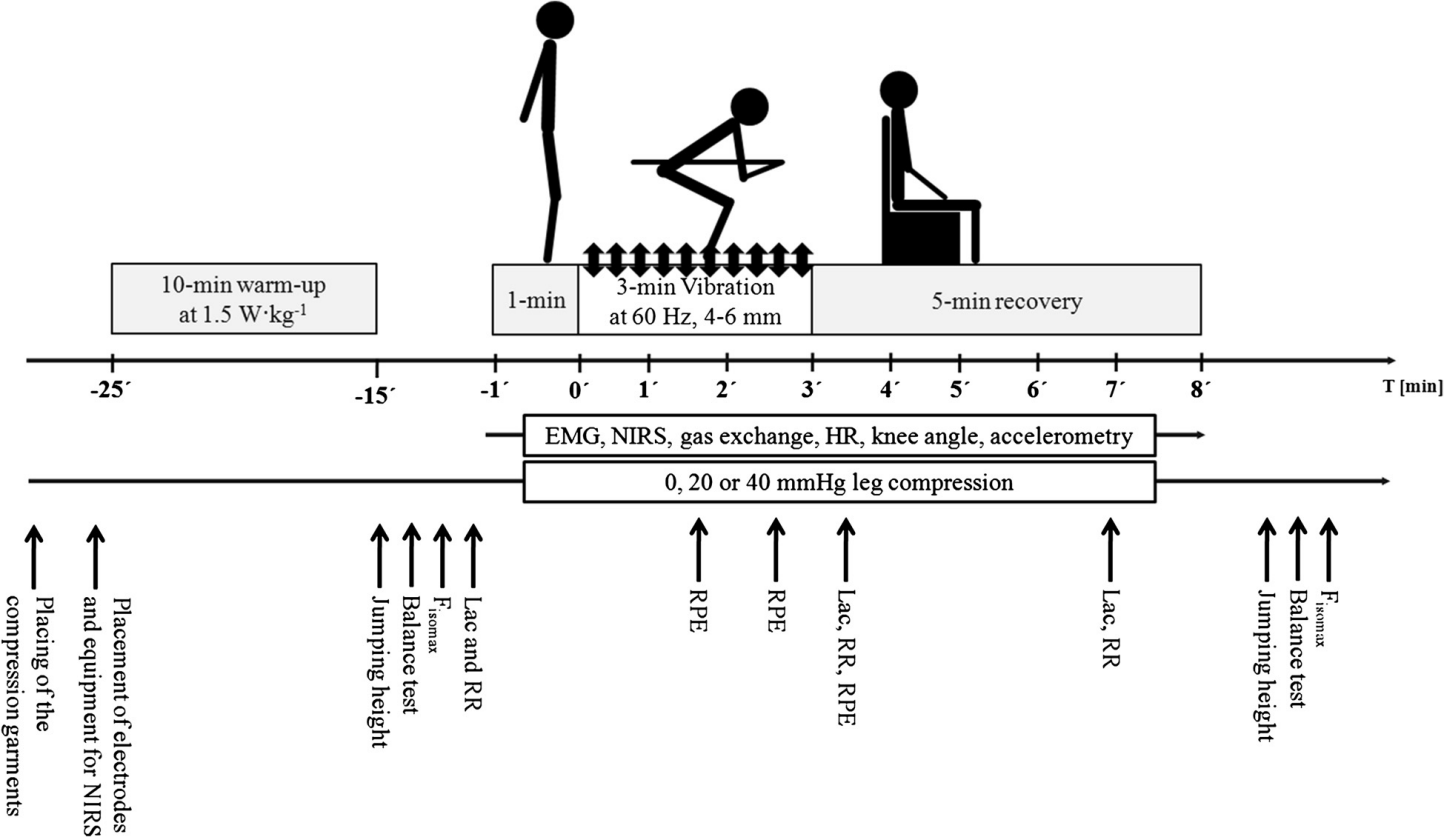
**Schematic illustration of the experimental design, indicating the time-points at which measurements were performed prior to, during and after taking the tuck position with vibration and different levels of compression (0, 20 and 40 mmHg).** NIRS = near-infrared spectroscopy, HR = heart rate, EMG = electromyography, Fisomax = maximal isometric force, Lac = blood level of lactate, RPE = rating of perceived exertion, BP = blood pressure.

The overall study design, including the time-points for data collection, is illustrated schematically in Figure 1.

To simulate actual alpine skiing, the 3-min trials in a downhill tuck position involved application of passive vibration to the soles of both feet by a Fitvibe® Excel Pro vibration platform (Vreden, Germany). Preliminary testing revealed that with application of passive vibration at 60 Hz and an amplitude of 6 mm, together with an additional load equivalent to 10% of body mass, three minutes (i.e., approximately the duration of an actual event) was the maximal time for which an experienced skier could remain in a downhill tuck position. Each participant performed three trials in random order, one each wearing leg garments that exerted no (0 mmHg), moderate (20 mmHg) or a high (40 mmHg) level of compression and separated by at least 2h of recovery. All athletes wore compression socks that extended from below the knee to the foot and compression shorts beginning at the knee and ending at the waistline (both from Sigvaris, Switzerland), as well as their own racing boots. The level of compression on each participant’s calf and thigh was checked five times before each test by the same procedure applied during preliminary testing.

The standard protocol began with 1 min standing in a relaxed position with no passive vibration, after which the subjects assumed their normal downhill skiing tuck position and maintained this position with no evasive movements throughout the 3 min of vibration (see Figure 1). Following each trial, each participant was allowed to recover for 5 min seated on a chair next to the vibration platform with his knees at an angle of 90°.

Prior to and after the 5-min exposure to passive vibration, counter-movement jumping height, balance and maximal isometric knee flexion and extension strength were tested, in that order. In addition, the following parameters were monitored before, during, and after all trials: the EMG activity of the *tibialis anterior, gastrocnemius medialis, rectus femoris, vastus lateralis and medialis, biceps femoris* and *gluteus maximus* muscles; oxygenation of the *vastus lateralis* muscle; gas exchange; heart rate; lactate concentration in the blood; and perceived exertion.

#### Testing isometric strength

The maximal voluntary isometric strength and rate of force development during extension and flexion of the knee muscles were determined utilizing a 5-kN force sensor (K Toyo 333A, Seoul, Korea). In the case of extension, the participants were seated with hip and knee angles of 90° and 120°, respectively: while for testing flexion, the knee angle was 160°. To minimize body movement, the participants were strapped to the seat with a belt secured around the hips during all tests. They were instructed to exert maximal force as quickly as possible against the force sensor (attached to the ankle with a Kevlar rope) and to maintain this force for 3 - 4 s. Standardized verbal encouragement was provided.

The greatest force exerted during three maximal voluntary contractions (MVC; performed with 1-min intervals of rest) was employed for statistical analysis. Trials during which an initial counter**-**movement (identified on the basis of a discernible drop in force preceding the rise) occurred were not included. The MVC, calculated as the average force registered during the 600-ms period centered around the moment of peak force, was used for normalization of the EMG. The height of counter-movement jumps (three trials with 1-min intervals of rest) was determined using a force platform with software specifically developed for this purpose (Accupower; AMTI, Watertown, Massachusetts, USA). This height was defined as the displacement of the center of mass, calculated from the force development and body mass.

### Measurement of balance

For assessment of balance, each subject stood on his dominant leg on a moving platform (Posturomed; Haider–Bioswing, Pullenreuth, Germany) with his hands on his hips and eyes closed. Balance was assessed as the period for which the participant could stand in this manner without opening his eyes, touching the security bar or putting down the other leg. This determination was performed 5 times.

#### Measurement of tissue saturation

Alterations in tissue concentrations of oxy- [HbO_2_], deoxy- [HHb] and total hemoglobin [tHb] were monitored with a portable near-infrared spectrophotometer (NIRS) (Portamon, Artinis Medical System, Zetten, the Netherlands) attached with a 10 x 10-cm adhesive patch across the widest girth of the left *vastus lateralis* muscle, as described previously [28]. The distance from the base of the patella along the vertical axis of the thigh to the center of this device was approximately 10 cm. The mean distance between the light source and optical detector was 3.0 cm and light with wavelengths of 760 and 840 nm was employed. To ensure that the thickness of the skin at the location of the NIRS device was less than half the distance between the source and the detector, skinfold thickness was measured with Harpenden calipers (British Indicators Ltd., West Sussex, UK) prior to all testing [29]. A marker allowed accurate repositioning of the sensor for each subsequent trial. The tissue saturation index (TSI, expressed as % and calculated as [HbO_2_]/([HbO_2_] + [HHB]) x 100) provided an indicator of the equilibrium between oxygen supply and consumption. Previous findings have demonstrated that the TSI provides a more accurate indication of muscle oxygenation than [HHb] [28,30]. The absolute TSI values observed at 30 s, 60 s, 120 s and 180 s into each trial were used for statistical analysis and the increases in these values following 15 s, 30 s, 60 s, 120 s and 300 s of recovery were taken as indices of the rate of re-oxygenation [28,31].

#### Measurement of electromyographic activity

For surface recording of the EMG activities of leg muscles (EMG; TeleMyo 2400T; Noraxon Inc., Scottsdale, AZ, USA), pre-gelled bipolar electrodes were placed on the muscle belly aligned parallel with the fibers, in accordance with international standards [32]. Prior to this, the surface of the skin was shaved, abraded slightly and cleansed with alcohol. All EMG signals were amplified differentially (Biovision, Wehrheim, Germany), filtered through a hardware band-pass (10-500 Hz at 3 dB), converted to digital units (DAQ 700 A/D card, National Instruments, Austin, TX, USA), sampled at 2 kHz, and analyzed with version 1.06.50 of the MyoResearch software (Noraxon Inc., Scottsdale, AZ, USA). Prior to further processing, all of these signals were then full-wave rectified. The mean EMG values for each window were calculated and the maximal amplitudes normalized to the corresponding MVC were calculated with the moving-window technique (stepwise value-for-value; window size, 250 ms). As an indicator of the activity of each muscle, the integrated EMG (iEMG) values were calculated from these normalized signals and subsequently expressed as a percentage of the MVC (%).

#### Measurement of knee joint angle

The angle of the knee joint was determined with a goniometer (potentiometers: Megatron, Munich, Germany; strain gauges: Penny & Giles Controls Ltd., Cmwfelinfach, UK) at 2000 Hz and expressed in degrees (°). This instrument was calibrated 5 times with the knee at angles of 90° and 180°, with alignment between the femur and tibia defining 180°. Angles were calculated from the corresponding mean voltage data.

#### Measurement of acceleration

Two triaxial accelerometers (Noraxon 3D accelerometer sensor, Noraxon U.S.A. Inc., Scottsdale, Arizona, USA) with a sampling frequency of 1500 Hz were used to monitor acceleration (g). One of these was placed on the *vastus lateralis* muscle and the other on the vibration platform, using adhesive tape in both cases.

#### Determination of blood lactate concentration

In order to compare the metabolic responses with the three types of clothing, blood samples were collected from the participant’s left earlobe into a capillary tube (Eppendorf AG, Hamburg, Germany). Blood lactate concentration was analyzed in duplicate by an amperometric-enzymatic procedure (Ebio Plus, Eppendorf AG, Hamburg, Germany) and the mean value utilized for statistical analysis. We chose to sample blood 4 min after the 3-min tuck position, since this is when blood lactate concentration after maximal exercise is highest, both with and without compression clothing [33].

#### Recording of respiratory data

Oxygen uptake, output of carbon dioxide, the respiratory exchange ratio, minute ventilation, and breathing frequency were assessed by a mixed expired procedure involving an Oxycon Pro apparatus (Jaeger GmbH, Hoechberg, Germany) equipped with an inspiratory flowmeter. The gas analyzers were calibrated with a high-precision two-component gas mixture containing 16.0% O_2_ and 4.0% CO_2_ (Air Liquide, Kungsängen, Sweden) and calibration of the flowmeter was performed at low, medium, and high flow rates with a 3-L air syringe (Hans Rudolph, Kansas City, MO, USA). Ambient conditions were monitored with an external apparatus (Vaisala PTU 200; Vaisala O**y**, Helsinki, Finland). The expired fractions of O_2_ and CO_2_ and the inspired minute ventilation (V_E_) were monitored continuously, all values being averaged every 20 s.

#### Measurement of heart rate and ratings of perceived exertion

Heart rate was monitored in 20-s intervals using a Polar telemetric system (Polar Wear Link System and Polar S810i HR monitor, Polar Electro Oy, Kempele, Finland). In addition, during the second minute of passive vibration and following the first, third and fifth minutes of recovery, the participants were asked to rate the perceived level of exertion for their whole body, thigh, and calf on Borg’s 6-20 scale [34].

### Statistical analyses

All analyses of descriptive data were performed using conventional procedures and the results are expressed as means and standard deviations (SD). When necessary, the effect size between pre- and post-testing was calculated for all variables using Cohen’s *d,* with the thresholds for small, moderate and large effects being set at 0.20, 0.50, and 0.80, respectively [35]. All data were shown to demonstrate a normal distribution and no further transformation was required. Repeated-measures analysis of variance (ANOVA) was used to compare each dependent variable at different time-points. When a global difference over time was observed, Bonferroni post-hoc analysis with correction for the effect of multiple comparisons was utilized as 0.05/n, where n is the number of comparisons. An alpha value of < 0.05 was considered to be statistically significant and all analyses were performed with the Statistica software package for Windows® (version 7.1, StatSoft Inc., Tulsa, OK, U.S.A).

## Results

The level of compression applied on the maximal girth of the calf and thigh by the racing suit and/or underwear during preliminary testing and by the compression garment during testing are documented in Table 1.

**Table 1:** **The level**s **of compression (mean ± SD) during preliminary (n = 7) and laboratory testing (n = 12)**

	**Preliminary test with racing suit**	**Laboratory testing with**	
		**Moderate compression (20 mmHg)**	**Strong compression (40 mmHg)**
Calf [mmHg]	13.5 ± 0.7	19.7 ± 3.7	39.5 ± 3.5
Thigh [mmHg]	11.4 ± 2.5	17.8 ± 1.9	34.0 ± 2.6

The maximal voluntary isometric strength and rate of force development during leg extension and flexion, as well as the counter-movement jump height and balance were all the same prior to and after exposure to passive vibration (Table 2, *P* > 0.05 in all cases; best effect size = 0.78).

**Table 2:** Leg extension and flexion, jumping performance and balance (means ± SD) prior to (pre) and following (post) 3 min in the downhill tuck position with vibration and different levels of compression

		**Compression [mmHg]**	**Pre**	**Post**	***P***	***d***
Leg extension	F_max_ [N]	0	501 ± 135	479 ± 96	>0.05	0.18
		20	489 ± 104	482 ± 96		0.06
		40	496 ± 90	486 ± 109		0.10
	Rate of force development [N·s^-1^]	0	1480 ± 485	1340 ± 571	>0.05	0.26
		20	1500 ± 475	1380 ± 608		0.23
		40	1500 ± 647	1560 ± 673		0.08
Leg flexion	F_max_ [N]	0	221 ± 48	212 ± 24	>0.05	0.23
		20	208 ± 34	197 ± 41		0.37
		40	197 ± 28	191 ± 32		0.19
	Rate of force development [N·s^-1^]	0	438 ± 102	351 ± 120	>0.05	0.78
		20	440 ± 170	374 ± 94		0.60
		40	467 ± 109	395 ± 137		0.58
Counter movement jump [cm]		0	36.7 ± 5.9	35.8 ± 7.2	>0.05	0.13
		20	36.4 ± 5.4	35.6 ± 6.0		0.14
		40	36.1 ± 6.4	37.1 ± 7.2		0.14
Balance time [s]		0	25.7 ± 21.6	27.4 ± 20.4	>0.05	0.08
		20	26.0 ± 19.4	25.9 ± 16.3		0.00
		40	17.9 ± 12.1	22.1 ± 16.35		0.29

The knee angles, acceleration and EMG recordings during the 3-min period in the alpine skiing tuck position with passive vibration are summarized in Table 3.

**Table 3:** Knee angles, absolute and integrated acceleration of the thigh muscles, and relative differences in EMG activation (means ± SD) during the first 30 s of the 3-min period in the downhill tuck position with vibration and different levels of compression

	**Compression [mmHg]**	**1**^**st **^**minute**	**2**^**nd **^**minute**	**3**^**rd **^**minute**
		**In the downhill tuck position**		
Knee angle [°]	0	123.9 ± 10.6^a,b^	123.0 ± 13.3^a,b^	121.2 ± 14.1^a,b^
	20	113.1 ± 8.3	113.0 ± 7.5	110.4 ± 9.2
	40	111.6 ± 9.2	110.4 ± 11.1	109.5 ± 12.5
Acceleration [g]	0	0.89 ± 0.29	0.95 ± 0.44^b^	0.98 ± 0.33^b^
	20	0.89 ± 0.37	0.85 ± 0.38	0.78 ± 0.41
	40	0.84 ± 0.29	0.79 ± 0.24	0.73 ± 0.16
Integrated acceleration [g·s]	0	26.7 ± 8.6	28.3 ± 13.1^a^	29.2 ± 15.8^a,b^
	20	26.7 ± 11.1	25.5 ± 11.3	23.1 ± 12.1
	40	25.1 ± 8.6	23.7 ± 7.3	22.0 ± 4.8
EMG [% MVC] of the muscle				
Tibialis anterior	0	75.9 ± 38.03	67.9 ± 40.2^a^	89.3 ± 75.5^a,b^
	20	69.4 ± 27.2	83.3 ± 71.5	125.6 ± 133.5
	40	82.1 ± 27.5	100.8 ± 92.3	112.0 ± 55.9
Gastrocnemius medialis	0	92.7 ± 18.1	83.2 ± 16.1	90.3 ± 15.1^b^
	20	95.9 ± 14.5	95.3 ± 24.3	95.0 ± 23.1
	40	89.4 ± 19.4	96.2 ± 25.8	106.4 ± 29.3
Rectus femoris	0	106.7 ± 17.8	114.4 ± 22.9^b^	136.5 ± 39.4^a,b^
	20	105.7 ± 23.9	120.6 ± 26.3	151.1 ± 43.6
	40	107.5 ± 16.8	144.6 ± 64.0	178.8 ± 92.2
Vastus lateralis	0	99.8 ± 6.3	101.2 ± 9.62	118.7 ± 27.2
	20	98.0 ± 4.2	103.7 ± 12.3	123.4 ± 23.4
	40	100.0 ± 4.0	106.9 ± 15.8	125.2 ± 23.9
Vastus medialis	0	101.3 ± 17.6	89.7 ± 20.5	100.7 ± 26.3^a,b^
	20	93.3 ± 5.8	96.7 ± 12.8	112.5 ± 26.6
	40	95.3 ± 7.3	99.2 ± 11.9	116.2 ± 20.6
Biceps femoris	0	90.2 ± 19.7	82.9 ± 16.2	97.2 ± 21.4
	20	89.3 ± 11.2	89.2 ± 19.1	104.3 ± 28.1
	40	93.0 ± 14.8	92.2 ± 21.8	106.4 ± 33.9
Gluteus maximus	0	101.5 ± 16.0^a,b^	140.5 ± 80.2	165.8 ± 129.1^a,b^
	20	111.6 ± 12.5	133.3 ± 43.7	134.0 ± 63.3
	40	114.0 ± 22.8	132.7 ± 52.5	134.0 ± 133.0

^a^*P* < 0.05 in comparison to the corresponding value with 20 mmHg.

^b^*P* < 0.05 in comparison to the corresponding value with 40 mmHg.

The knee angle during the 3-min period in the tuck position was approximately 10° less when subjected to 20 or 40 mmHg compression than with no compression at all (*P* < 0.01; effect sizes 0.82 – 0.90). Both absolute and integrated acceleration were 20 – 25.5% and 26.0 – 32.0% lower, respectively, during the second and third minutes of passive vibration with compression than without (*P* < 0.01; effect size = 0.61 – 0.96). During the third minute of passive vibration, the EMG activities of the *tibialis anterior* (−20.2 – 28.9%), *gastrocnemius medialis* (−4.9 – 15.1%), *rectus femoris* (−9.6 – 23.5%) and *vastus medialis* (−13.1 – 13.7%) were lower (*P* < 0.01 in both cases; effect size = 0.25 – 1.78), whereas the corresponding activity of the *gluteus maximus* was significantly higher (+ 23%) (*P* < 0.01 in all cases; effect size = 0.16 – 0.99) in the absence of any compression.

**Figure 2:**
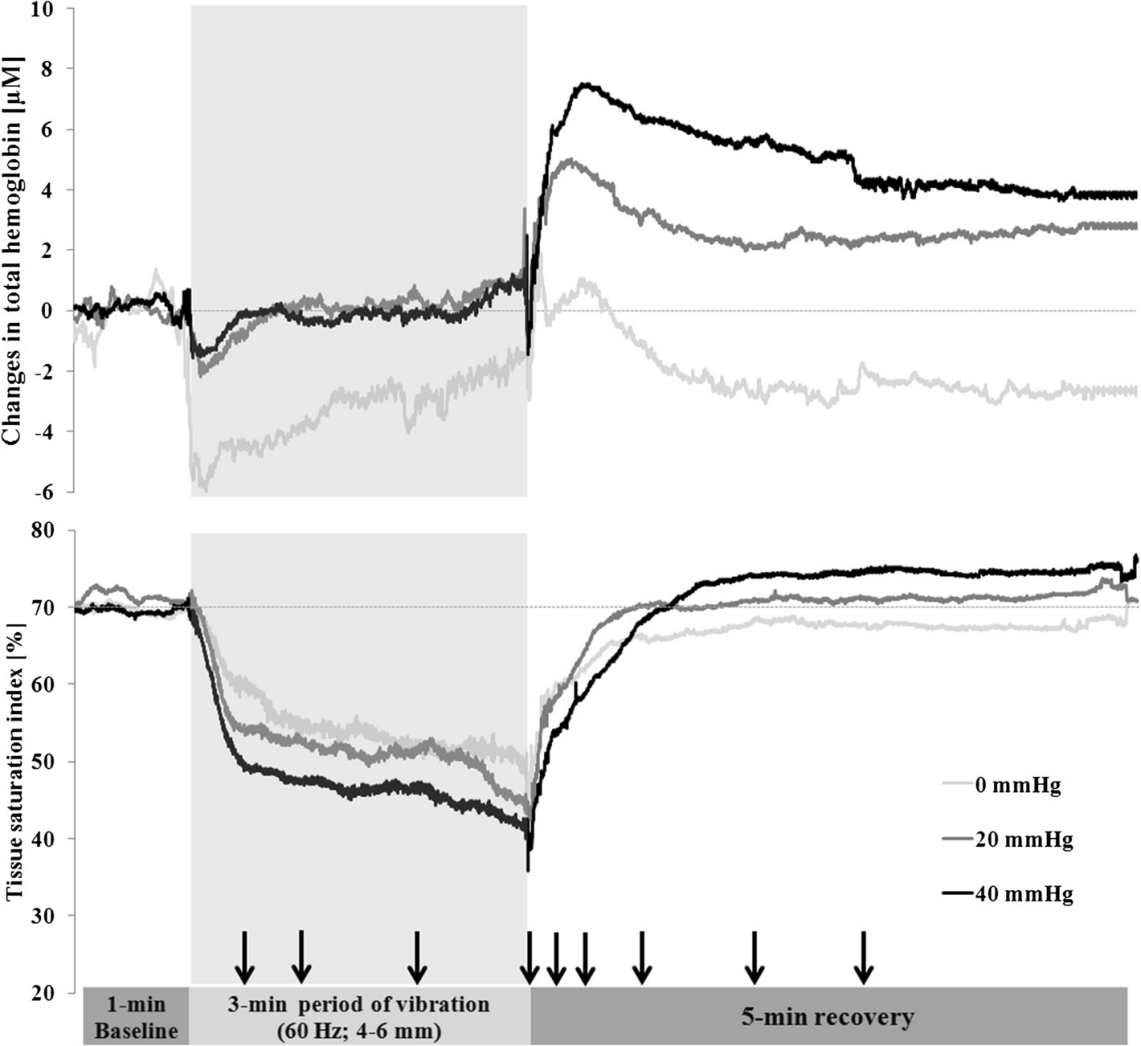
**Mean change (n = 12) in the total hemoglobin concentration and tissue saturation index prior to, during and following 3 min in the downhill tuck position with vibration and different levels of compression.** For the sake of clarity, we have not illustrated the standard deviations, which were at similar magnitudes with all clothing. The arrows indicate the time-points at which the data collected was subjected to statistical analysis.

Immediately after being subjected to passive vibration for 3 min with compression, the tissue saturation index was lower than without compression (Figure 2; *P* < 0.01; effect sizes = 1.16 – 1.75). At the same time, reoxygenation after approximately 90s of recovery was enhanced significantly by compression (*P* < 0.01 in all cases; effect size = 1.64 – 13.60; Figure 2). Total hemoglobin during the 3-min period in the tuck position was significantly lower without compression than with 20 or 40 mmHg of leg compression (*P* < 0.01; effect size = 1.64 – 2.07; Figure 2).

**Figure 3:**
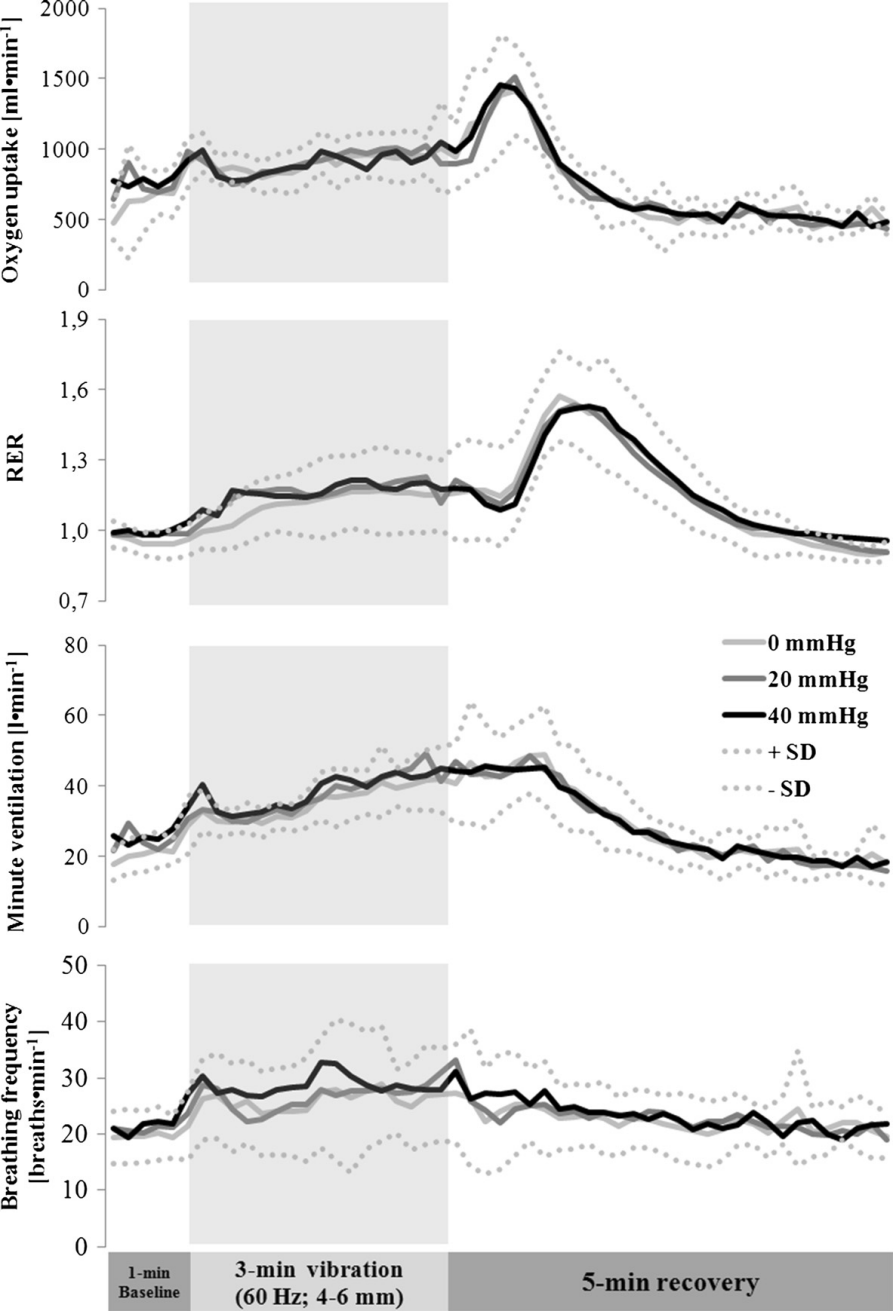
**Oxygen uptake, the respiratory exchange ratio (RER), minute ventilation and breathing frequency prior to, during and following 3 min in the downhill tuck position with vibration and different levels of compression.** Mean values and SDs (n = 12) are shown.

As illustrated in Figure 3, oxygen uptake, the respiratory exchange ratio (RER), minute ventilation and breathing frequency were the same at all time-points with and without compression (*P* > 0.05). Oxygen uptake peaked (1410 ± 215 mL·min^-1^) during early recovery. The peak values for RER, minute ventilation and breathing frequency ranged from 1.54 – 1.56 and 48.9 – 49.2 L·min^-1^ and 31 – 32 min^-1^, respectively.

As documented in Table 4, blood lactate levels and heart rate with the different levels of compression did not differ at any time. However, with either 20 or 40 mmHg compression, perceived exertions for the whole body, thigh and calf were lower than in the absence of compression (*P* < 0.05; effect size = 0.75 – 0.90).

**Table 4:** Blood lactate concentration, heart rate and perceived exertion (means ± SD) prior to, during and following the 3-min period in the downhill tuck position with vibration and different levels of compression

		**Prior to the test**	**Period of vibration**			**Period of recovery**				
**Parameter**	**Compression [mmHg]**		**1**^**st **^**minute**	**2**^**nd **^**minute**	**3**^**nd **^**minute**	**1**^**st **^**minute**	**2**^**nd **^**minute**	**3**^**nd **^**minute**	**4**^**th **^**minute**	**5**^**th **^**minute**
Blood lactate concentration [mmol·L^-1^]	0	1.25 ± 0.33				2.80 ± 0.67	4.37 ± 1.02	4.68 ± 1.34		4.39 ± 1.33
	20	1.32 ± 0.22		not determined		2.60 ± 0.47	4.04 ± 0.77	4.46 ± 1.00		4.19 ± 1.12
	40	1.22 ± 0.24				3.06 ± 0.63	4.11 ± 0.75	4.70 ± 1.36		4.31 ± 1.26
Heart rate [beats · min^-1^]	0	78 ± 9	123 ± 18	137 ± 25	137 ± 26		113 ± 22	88 ± 21		78 ± 14
	20	77 ± 12	126 ± 18	140 ± 21	142 ± 23		108 ± 20	86 ± 16		79 ± 12
	40	79 ± 11	128 ± 21	139 ± 25	138 ± 26		104 ± 25	85 ± 14		78 ± 14
Rating of perceived exertion [score on Borg’s scale] Whole body	0			14.2 ±1.6^a,b^		14.4 ± 1.4		11.5 ± 1.6		9.4 ± 1.8
	20			12.8 ±1.4		14.5 ±1.7		11.2 ±2.6		9.5 ± 2.2
	40			13.4 ±1.5		14.1 ±1.9		11.1 ± 2.5		9.5 ± 2.3
Thigh	0			16.3 ± 1.2^a,b^		17.5 ± 1.6		13.4 ± 2.3		11.6 ± 2.3
	20			15.5 ± 1.5		17.4 ± 1.8		13.5 ± 2.7		11.8 ± 2.6
	40			15.4 ± 1.2		16.9 ± 2.2		13.5 ± 2.6		11.5 ± 2.8
Calf	0			12.8 ± 1.9^a,b^		13.7 ± 2.7		10.6 ± 2.1		9.8 ± 1.9
	20			11.5 ± 2.1		13.8 ± 1.9		10.9 ± 2.8		8.9 ± 2.5
	40			11.3 ± 2.8		12.9 ± 2.9		10.4 ± 2.6		9.4 ± 1.9

^a^*P* < 0.05 in comparison to the corresponding value for 20 mmHg.

^b^*P* < 0.05 in comparison to the corresponding value for 40 mmHg.

## Discussion

Our major findings concerning the effects of compression garments during a 3-min period of simulated alpine skiing in the tuck position accompanied by passive vibration were as follows: 1) the knee angle was lowered by approximately 10°; 2) oscillatory movement was reduced by 20–25.5%; 3) perceived whole body, thigh and calf exertion were reduced; 4) activity in most of the associated muscles was elevated; 5) the de- and reoxygenation of the *vastus lateralis* muscle were both more rapid; 6) gas exchange parameters, blood lactate concentration and heart rate were unaffected; and 7) compression did not exert any effect on the maximal voluntary isometric strength of knee flexors and extensors, the rate of force development during knee extension and flexion, counter-movement jumping height or balance.

Judging from the reduction in knee angle and oscillatory movement of the thigh muscles, 20 or 40 mmHg of compression led to a deeper tuck position, with less transmission of vibration to the thigh. From an aerodynamic point of view, a lower, more compact tuck position should attenuate air drag, thereby enhancing speed [36,37]. Moreover, with both levels of compression, the participants experienced the exertion by their entire body, thighs and calves as less than in the absence of compression, i.e., they experienced the deeper position as less strenuous. Accordingly, compression garments may benefit alpine skiers by reducing vibration, air drag and lowering perceived exertion.

It is well known that the primary afferent endings of muscle spindles are stimulated by passive vibration [38,39], activating, in turn, a large number of motoneurons [5].Thus, attenuation of the oscillatory displacement of a muscle subjected to passive vibration while in the tucked position should attenuate activation of muscle fibers. Here, however, this was only the case for the *gluteus maximus*, the activity of which was reduced 23% by compression. All of the other muscles monitored, i.e., the *tibialis anterior* (+ 20.2 – 28.9%), *gastrocnemius medialis* (+ 4.9 – 15.1%), *rectus femoris* (+ 9.6 – 23.5%) and *vastus medialis* (+ 13.1 – 13.7%), exhibited significantly higher activation with compression**,** possibly due to activation of muscle spindles via the polysynaptic vibration reflex [3,4].

Moreover, despite the structural support provided by the garment, compression caused our skiers to maintain a deeper tuck position, which should account for part of the enhanced EMG activity in most of the muscles. Furthermore, activation of the afferents of cutaneous tactile mechanoreceptors reduces presynaptic inhibition [40], which could also have elevated EMG activity.

Stretching of extra-dermal muscles has been reported to activate cutaneous receptors that transmit essential information concerning joint position to the central nervous system [40,41]. Moreover, stretching of the skin is a significant source of proprioceptive information [42]. Thus, application of compression might enhance joint proprioception, thereby improving balance and/or jumping. However, no such effects were seen here. The only published investigation concerning the influence of compression on balance that we could find, was the recent report that the average jumping height for members of a university track team was improved 5.2% by compression clothing [9]. The results documented here indicate that the balance and jumping performance of elite alpine skiers following a 3-min period of simulated skiing in a tucked position with passive vibration is not improved by compression.

Although muscle oscillation induced by passive vibration clearly enhances cardiovascular and metabolic demands [4,43,44], the concomitant impact on tissue oxygenation due to reduced skeletal muscle fatigue is controversial. In this context, oxygenation of the *vastus lateralis* muscle during 3-min of leg squatting with passive vibration at 15 Hz and 5 mm amplitude [45], as well as the rate of oxygen utilization by the *gastrocnemius medialis* muscle during heel lifting at 16 Hz and 4 mm amplitude [46] were higher than in the absence of vibration, as determined by near-infrared spectroscopy (NIRS). In contrast, NIRS oxygenation of the *vastus lateralis* and *gastrocnemius medialis* muscles in sedentary and physically active males assuming the static squat-position was not affected by vibration at frequencies of 30, 40, and 50 Hz and an amplitude of 4 mm [47]. Finally, oxygenation of haemoglobin (determined by magnetic resonance spectroscopy and NIRS) during 3 minutes of isometric plantar flexion exercise at 40% of maximal voluntary contraction was not influenced by passive vibration (20 Hz and 2 mm amplitude) [48].

Our NIRS data indicate that the *vastus lateralis* muscle was more severely deoxygenated when exposed to 40 mmHg of compression than with 20 mmHg or no compression. As described previously [45], the level of oxygenation in human skeletal muscle reflects the balance between oxygen supply and utilization [49,50]. Thus, this attenuated oxygenation of the *vastus lateralis* muscle at 40 mmHg of compression may reflect either attenuated oxygen supply and/or elevated oxygen utilization. A recent study in which positron emission tomography (PET) was employed to monitor muscle metabolism following 30 min of high-intensity cycling with a high level of compression (~37 mmHg) revealed reduced blood flow in both the deep and superficial regions of the *m. biceps* and *m. quadriceps femoris* during recovery [51]. Although the methodology and conditions used in this other and the present investigation differ, it appears likely that the high level of compression employed here (40 mmHg) reduced blood flow and thereby diminished the supply of oxygen to the working muscle. In addition, in our study the knee angle during 3 min in the tuck position with compression was approximately 10° lower than without compression and the EMG activity of the *vastus lateralis* muscle was 7% higher. We therefore conclude that the more intense muscle activation caused by this smaller knee angle in combination with reduced blood flow was responsible for the increased deoxygenation associated with 20 or 40 mmHg of compression.

Oxygen uptake and blood levels of lactate are informative parameters, since the supply of oxygen to working muscles is considered to be a limiting factor with respect to performance [52,53]. Both of these parameters, as well as the heart rate and respiratory exchange ratio, were uninfluenced by the type of clothing worn, even though the *vastus lateralis* muscle was more highly deoxygenated with compression (see above). Since measurement of NIRS reflects the local balance between oxygen supply and utilization [49,50], which in turn, is an integrated indicator of oxidative metabolism, it would seem that the compression clothing worn here did not alter whole-body oxidative metabolism.

Several other investigations have concluded that compression clothing reduces the concentration of lactate in the blood following exercise [54,55], whereas the largest effect sizes observed here were moderate (0.18-0.25), at best. The observation by Berry and McMurray [55] that blood lactate concentrations were reduced during recovery from a 5-min maximal-effort treadmill test through the use of compression stockings was later confirmed by Chatard and co-workers [54]. The former investigators state that the lower concentrations they detected could not be attributed to shifts in plasma volume, but appeared to be due to creation of an inverse gradient by the stockings, resulting in retention of lactate in the muscular bed. However, when examining the kinetics of blood lactate during high-intensity cycling and recovery, Rimaud and colleagues [33] concluded that compression promoted clearance of blood lactate to a limited extent only. In light of these findings, it remains unclear whether compression garments influence the release of blood lactate into or removal of lactate from the blood following high-intensity exercise.

### Limitations of the present study

Our standardized and well-controlled laboratory conditions are, of course, different and, indeed, somewhat artificial in comparison to actual outdoor skiing, where velocity, snow conditions, skis and level of skill may all exert a significant impact on the vibrations that arise. With field testing on a ski slope, it is not possible to consistently reproduce periodic, sinusoidal oscillatory motions of exactly the same magnitude and degree of randomization [2]. Therefore, we chose to simulate constant passive vibration in the laboratory to optimize standardization and to ensure that the frequency of vibration remained well within the range actually encountered during alpine skiing [2,24]. Indeed, our participants confirmed that the vibration they were subjected to was similar to what they experience during alpine skiing. Finally, participant fatigue might influence reproducibility between the three test trials in a study such as the present one. To minimize this confounder, we recruited only highly skilled skiers with experience of national and international competition and the ability to ski efficiently in a tucked position.

## Conclusions

Compression on the legs of elite alpine skiers performing simulated skiing for 3 min in the tucked position with passive vibration resulted in a deeper tuck position with a lower level of perceived exertion, with greater deoxygenation of the *vastus lateralis* muscle but no differences in whole-body oxygen consumption or blood lactate concentration. These changes occurred without compromising maximal leg strength, jumping performance or balance. Accordingly, our findings indicate that the use of lower leg compression in the range of 20–40 mmHg may improve alpine skiing performance due to a deeper tuck position and lower perceived exertion.

## Competing interests

The study was funded by our own institutional resources. The clothing was provided by Sigvaris, (Winterhur, Switzerland), who played no role in study design, data collection and analysis, the decision to publish, or preparation of the manuscript. No additional external funding was required or received. BS has received a consultancy fee from SIGVARIS in connection with earlier projects, but not the present one. None of the authors owns stocks and is employed by or sits on the board of SIGVARIS AG or any other company with competing financial, professional, nor personal interests in relationship to the data presented here.

## Authors’ contributions

Conception and design of the experiments: BS DPB MS YK BG HCH. Performance of the experiments: BS DPB MS YK BG. Analyzed the data: BS DPB MS YK BG MKB HCH. Provision of reagents/materials/analytical tools: BS DPB MS YK BG MKB HCH. Preparation of the manuscript: BS DPB MS YK BG MKB HCH. All authors read and approved the final manuscript.

## Pre-publication history

The pre-publication history for this paper can be accessed here:

http://www.biomedcentral.com/2052-1847/5/18/prepub
